# A Consideration of the Comparative Merits of Watts’ Crystal Gold among Filling Materials

**Published:** 1884-11

**Authors:** J. F. P. Hodson

**Affiliations:** New York


### A CONSIDERATION OF THE COMPARATIVE MERITS OF WATTS' CRYSTAL GOLD AMONG FILLING MATERIALS.

READ BEFORE THE NEW ENGLAND DENTAL SOCIETY, BOSTON, OCT. 3, 1884.

BY J. F. P. HODSON, D. D. S., NEW YORK.

It is with much diffidence, and yet with a lively sense of the honor you have conferred upon me in extending to me the special and very kindly invitation to appear before you, that I am here to-day, and while thanking you for the distinguished courtesy of which I am the object, I cannot but feel how much more you might have bettered the selection of your object than your subject. There are few, if indeed there be any, subjects that could more properly engage our most serious consideration than the comparative merits of filling materials for the teeth, as few I think will question that whatever our surgical or other dental skill or abilities, the perfect stopping is, in a very real sense, the *ultima thule* of all dentistry. As, however, it has been very delicately hinted to me by your committee that your especial hunger is for practical facts rather than theoretical disquisition, I will plunge at once into the midst of them by the introductory premise that "Watts' crystal gold" is not to be confounded with any of the other so-called crystal golds that are offered us from time to time. It is, as is clearly shown under the microscope, an interlaced mass of perfect golden fern leaves, that are deposited from and in the continuously saturated solution of gold by a process of electrical action, while all others that I have ever examined show no organization whatever, but are a mere agglomerated mass of gold particles which cohere temporarily like wet sand, and afterward disintegrate like dry, and are, moreover, produced by chemical action, mercury, etc., etc., methods which were experimented with and abandoned by Watts twenty-five years ago.

I have used this beautiful form of gold exclusively for something more than fifteen years, and associated with other golds before that time, and so affirm but what I know when I state that all operations which can be accomplished with any other gold or by any operator can be as perfectly accomplished with this, and with less expenditure of force and strain, and, indeed, in every sense far greater ease of manipulation than with foil golds. Crystal gold, moreover, possesses some excellencies which do not obtain in foils, one of which is the steel-like hardness of surface of the fillings produced with it, which in grinding surfaces is so especially valuable for their resistance to and withstanding of the battering force of mastication and of the cusps of opposing teeth, and in the exquisite perfection with which it adapts itself—*under honest manipulation*—to the walls of the cavity. Indeed, so close and perfect is this that the delicate pointings of the microscopic crystals have been shown to have entered the open ends of the dentinal tubuli themselves. This waxy adaptiveness would be under any circumstances a most valuable attribute, but it becomes doubly so when taken in connection with the fact that the gold is condensed with so little force as compared with foil. In such circumstances, for instance, as very frail front teeth, where, for the sake of appearance, it is often expedient to save every atom of even thin remaining enamel, the value of this quality in crystal gold is inestimable. I wish to be perfectly understood with regard to the condensation of crystal gold in these instances. The filling should be, and of course is, with faithful manipulation of this gold with small points, practically as closely adapted and as well consolidated against those walls and throughout the stopping as if against strong walls, and all this where a proper condensation of foil would be far more likely to rupture the enamel, even granting to the foil the most skillful manipulation. If crystal gold can be depended upon to do such delicate and refined work among "egg-shells," I deem it a work of supererogation to enlarge upon, or even do more than suggest the point to intelligent professional gentlemen, what must be its capacity for easy and perfect adaptation in the great mass of the usual cavities having strong walls. This wonderful combination of all the compliant and waxy characteristics of "soft" foil with even more of the cohesive quality than is possessed by most cohesive golds, and superadded to this a very hard finished surface peculiarly its own, are the especial characteristics of Watts' crystal gold.

As I have previously said, I have used this form of gold exclusively for more than fifteen years, and although I always have a quantity of other golds in my gold drawer and obtain most of the new varieties as they appear, keeping them more or less even on my trays, because I despise the prejudiced and obstinate riding of a hobby, and always feel ready to use any gold or any material with which I can produce the best results in each and any individual case, I am nevertheless wholly unable, as a general rule of every-day practice, to find any place in any cavity where I can possibly employ them to any advantage as compared with crystal gold, and am constrained to store away the pellets and cylinders, etc., in the body of large stoppings from time to time, *to get rid of them.* Aha! I hear some captious critic say, "So you *do* manage practically to combine foil with your crystal gold, after all." By no means, gentlemen. I merely seek to dispose of the, to me, useless material by putting it where possibly many of you would say "it would do the most good," but where I would say it could do the least harm, in the center of a large stopping, precisely as I would stow away pieces of crystal gold that had become in any way somewhat condensed, and so, in my opinion, unfitted to place next the walls.

One exception, however, to my exclusive employment of crystal gold that I wish to mention is, as a mere matter of extra prudence, my habit of binding together a largely built-out restoration in gold with occasional narrow strips of heavy foil, feeling that the magnificent hardness of crystal gold might possibly militate against the toughness and consequent integrity of a long, thin restoration. Besides this negative proposition, as to my employing no other gold, I wish to place the positive one that, so far from employing it only for usual operations and then plastering up all the difficult or large ones with amalgam, I almost never (probably not nearly as often as I ought) employ amalgam or other plastic filling materials for permanent teeth, and as seldom order teeth out. I say all this in order to make the point, and to make it plain, that I *never*

*hesitate about doing any operation whatever with crystal gold*, that I depend upon it for all and any dental stoppings or restorations whatsoever, small and great, easy and difficult, and that if any failure occurs (and I am not, I hasten to assure you, one of those transcendent operators who never, never have them), I know the failure to be consequent upon my somehow or somewhere defective manipulation, and not any fault of the gold employed. For instance, if I were to "take a piece as large as the cavity" (as for many years the instructions accompanying it directed, and as some operators who use it apparently do now) and jam and plaster it into the cavity, trusting to some magic of affection in the gold to seek out the walls and cling to them, I should fully expect, as a consequence, the leaky and worthless stopping that is so often seen, and which advertises to all who see it, not the incompetency of the gold, but of the operator who essayed to place it. Notwithstanding the beautiful, even silky, softness and pliancy of crystal gold as it takes its place in the cavity, it becomes hard under manipulation, and that very quickly, and being cohesive to the last degree must be treated, after this first placing in the cavity, like cohesive foil; and as I have said in a previous paper, if crystal gold be given the same honest care that a conscientious operator would, in like circumstances, apply to cohesive foil, he would surely produce the same perfect stopping, and in addition receive the benefit of the grand combination of the special beauties and advantages contained in *both* soft and cohesive golds.

It is absolutely imperative that the first pieces of crystal gold be held immovably in the position in which they have at first been placed, and retaining points, at an angle of the cavity, may be employed for the purpose. My own very decided preference, however, and the method which I employ wherever possible, is to arrange to hold the completed stopping by the general shape of the cavity, rather than by means of the deep pits so often employed, as I greatly deprecate any unnecessary reaching out toward the pulp with conductive material. The first pieces may be held accurately to their first position by means of a pointed instrument in the left hand, continuing its employment until in condensing these first pieces and proceeding with the stopping, the shape of the cavity is found to hold the gold securely without it, condensing each piece finally as one progresses, exactly as a careful operator would proceed with cohesive foil, for, as I have before remarked, crystal gold *when first applied to the cavity* is waxy and adaptive as is the softest of soft foils, but there this similarity ends, as it should. This first application being once made, it must be treated thereafter precisely as though it were cohesive foil. A good operator when applying a cohesive foil pellet to the cavity would not proceed to disintegrate it by punching it full of holes with a sharp point. Neither would he, on the other hand, attempt to model large, thick pieces of it into position with "spoon" modelers, and expect anything but a worthless, uncondensed cavity, full of something akin to loose shavings or sheet-iron scraps as a result. Neither would he jam a large mass of cohesive foil into a cavity, and by thrusting wedge-shaped instruments down through it expect to force it to spread out sidewise and form the perfect adaptation to the walls of the cavity which the merest professional child ought to know is the *sine qua non* to the saving stopping. And yet operators do all of these things, commit all of these sins against crystal gold, and then shower maledictions upon the material for the results. "You miserable lamb," says the wolf, standing in the stream above him, "how you have roiled up and spoiled this water for me; I will destroy you." If operators would but take the cake of gold carefully and gently between the fingers of their left hand, and with long pointed tweezers in their right as carefully push off without too close approximation of the points of the tweezers a trayful of small pieces, and then, the rubber dam being in position on the tooth, the cavity dry, etc., take up one of the pieces on the end of such filling instrument as would be employed were cohesive foil pellets used, carry it through the annealing flame and to the position in the cavity where it is designed to commence the stopping, hold it absolutely still until it has been fully and entirely condensed, employing any means of condensation that would properly consolidate pellets of cohesive foil, add other pieces in the same manner, still holding the compacted portion immovably until it will, of a certainty, hold itself, adding no single piece, however, till that already in the cavity has been fully and finally consolidated, never changing the position of any pellet after its first application to its place, using so small pieces as to be *sure* that no piece is added which, from its size or thickness, they could not with absolute certainty condense throughout its substance without chopping or punching it full of holes, packing directly against the walls of the cavity, and remembering that no cohesive gold spreads or wedges out under manipulation like soft foil, and that, consequently, all packing must be done directly upon the part desired to be compacted — in a word, proceeding throughout the operation precisely as though it were cohesive foil pellets which were being employed — my word for it they would be surprised and delighted to find how they would be accomplishing the same results with this gold as with foil, and with how much greater ease and pleasure and delicacy they were doing it.

If they continued to the end of the placing of the gold, and polished and finely finished the exposed surface, they would find in that such resisting, steel-like hardness as is implied in the fact that a patient will return days afterward to have a mere nothing taken off from the surface. My own preference has always been for No. 1, in contradistinction to the more condensed Nos. 2, 3, and 4, and for such delicate handling of even this as that it shall certainly *be* No. 1 when it reaches the cavity, and not have been handled and pressed into the equivalent of Nos. 3 and 4, as the very essence of the value of crystal gold, to my mind, lies in the great difference in bulk between the exquisitely soft, silky state in which it comes to us, and its finally consolidated one in the cavity, and that this great change taking place under the guidance of the instrument of the operator implies all of the possibilities of the marvelously close adaptation to the walls of the cavity of which I have spoken, but which regal attribute of crystal gold is deliberately thrown away by using the condensed Nos. 2, 3, and 4, and equally so by the careless handling and squeezing of No. 1. In short, I want to do all of the condensing *in the cavity.*

The ultimate end desired by us all, with our varied methods and golds, is the goal of the perfect stopping or restoration, and each being devoted to his own method and becoming skillful in it, it becomes *his* best one, and we may learn much from these specialists, even though we ourselves may either not be specialists, or even do not at all employ his particular method.

The soft foil operator teaches us much about adaptation. The heavy foil man perhaps the want of it. The cylinder operator rapidity of operating. The electric foil man lauds its lead-like ductility. The cohesive foil man his general power to restore beyond the cavity. *Chacun a son gout.* But it is obviously unfair for one man to test another's method or material by his own manipulation, and then depreciate Pegasus because he won't yoke with the ox!

There are many different ways of getting from New York to Liverpool, and barring accidents one reaches there certainly by any one of the many lines; but as between them one's comfort and pleasure on the way and his condition upon arrival there are questions upon which each will have his own opinions and predilections; but supposing one to have been long accustomed to the cabins of a "Cunarder" or an "Inman" in his passages across, and upon the representations of a "White Star" patron decides to try, say the Germanic; it is certainly very unhandsome in him, not to say ridiculous, after having heedlessly stumbled down into the hold at the beginning of the voyage and as heedlessly remained there to the end of it, to go stamping about Liverpool upon his arrival out exclaiming against the manifold discomforts of the White Star Line and his passage, complaining that he was rolled and tumbled about, cut and bruised, until he was "all broken up," indignantly repudiating a suggestion as to his being intoxicated, and denying that he had been in that, to him, detestable condition from the first, declaring that he was indeed so very badly off that he must now go into the hospital to be made over, and that if there was any one thing sharply defined in his mind it was that he'd nevermore use that means for getting to Liverpool again.

"What's that you say—elegant promenade deck, and superb dining and sleeping cabins, reading room, smoking room, and all that? I don't believe it; I never found 'em." Surely such a passenger as this is quite as competent to judge between competing ocean steamers as is the operator who has bunglingly and unintelligently tested crystal gold by either exclusively soft or cohesive gold manipulations, to speak of its comparative merits.

